# TSH lowering effects of metformin: a possible mechanism of action

**DOI:** 10.1007/s40618-020-01445-9

**Published:** 2020-10-14

**Authors:** R. Cannarella, R. A. Condorelli, F. Barbagallo, A. Aversa, A. E. Calogero, S. La Vignera

**Affiliations:** 1 Department of Clinical and Experimental Medicine, University of Catania, Via S. Sofia 78, 95123 Catania, Italy; 2 Department of Experimental and Clinical Medicine, “Magna Graecia” University, Catanzaro, Italy

**Keywords:** TSH, Metformin, Hypothyroidism, Insulin-resistance, Thyroid, Insulin

## Abstract

Preliminary clinical evidence suggests that metformin has TSH lowering effects in patients with T2DM and hypothyroidism or in those with TSH serum levels in the upper normal value. Also, metformin may exert a protective role against thyroid nodules growth in patients without insulin-resistance. The cross-talk between tyrosine kinase receptors and the G protein-coupled receptors (which the TSHR belongs to) has been already shown and IRS1 may represent the hub link between TSHR and IR pathways. By influencing IRS1 phosphorylation pattern, metformin may sensitize TSHR to TSH, thus explaining the findings of clinical studies. However, the existence of this molecular pathway must be confirmed through proper studies and further prospective randomized placebo-controlled studies are needed to confirm this hypothesis.

Thyroid disease, obesity and type II diabetes mellitus (T2DM) represent the more common endocrine disorders. They are often concomitantly present in the same patient. Particularly, the prevalence of hypothyroidism in patients with T2DM is about 10–15% [1, 2]. Thus, the prescription of insulin-sensitizing drugs and, first of all, of metformin, is not infrequent among patients with hypothyroidism, goiter and thyroid nodules. This has led to consider the effects of metformin on thyroid disorders, including serum thyroid stimulating hormone (TSH) and free thyroxine (FT4) levels and thyroid nodules, resulting in the publication of a relevant number of studies in the last two decades. We used the key-words “metformin” and “TSH lowering effects”, to retrieve articles providing us with data useful to clarify the relationship between metformin and thyroid function.

In a prospective study-design, 101 patients with T2DM were treated with metformin. Among these, 29 hypothyroid patients were treated with levo-thyroxine (LT4), 18 hypothyroid patients did not receive LT4 and 54 were euthyroid patients. After 1 year of metformin administration, a significant decrease in TSH serum levels was reported in patients with T2DM and hypothyroidism (*n* = 47). No change was found in euthyroid patients. Furthermore, serum FT4 levels were not affected. Interestingly, the body mass index (BMI) did not differ following metformin administration, thus excluding a role for body weight decrease in the TSH lowering effect of metformin, especially in LT4 treated patients [3]. In the same study, a short-term administration of metformin (up to 24 weeks) did not affect TSH serum levels in 11 patients with T2DM and hypothyroidism receiving LT4 [3]. The TSH lowering effects of metformin have been firstly reported by Vigersky and coll.[4], and were following confirmed by Isidro and coll.[5], although in a limited number of patients. Particularly, in four patients with chronic hypothyroidism and not on LT4, metformin administration resulted in TSH suppression to subnormal levels, with no sign of hyperthyroidism [4]. A randomized controlled trial carried out in 60 patients with subclinical hypothyroidism reported no significant change in TSH and thyroid hormones serum levels between patients treated with metformin (1500 g daily) or receiving no treatment. However, the rate of normalization of TSH was significantly higher in treated patients with negative thyroid antibody compared to those who were antibody-positive [6], suggesting a TSH-lowering effect in patients who were thyroid antibody-negative.

In euthyroid patients with T2DM, metformin seems to lower serum TSH levels in those with values in the upper quartile. Accordingly, a retrospective study on 250 euthyroid patients with T2DM failed to confirm the TSH lowering effect of metformin. However, TSH values were not analyzed according to baseline quartile [7]. By contrast, a retrospective study on 393 euthyroid patients with T2DM divided the patients in 3 groups: the first did not receive neither metformin nor LT4 (*n* = 119); the second group received only metformin (*n* = 203); the third group received both metformin and LT4 (*n* = 71). Treatment was prescribed for at least 1 year. The results showed a significant decrease of serum TSH levels, independently of pre-treatment values, in the third group, which received LT4 at replacement doses. A significant reduction of serum TSH levels was observed also in euthyroid patients with high-normal pre-treatment TSH values (from 3.24 ± 0.51 to 2.27 ± 1.28 IU/l) belonging to the second group, which did not take LT4 [8]. At the multivariate regression analysis, these findings were independent from the BMI and from the presence of thyroid peroxidase antibodies. Furthermore, no change of TSH levels were observed in patients of the first group, which did not receive either metformin or LT4 [8]. Finally, a recent meta-analysis of 6 randomized controlled clinical trials including 494 euthyroid patients confirmed the TSH lowering effect of metformin after 1 year of treatment but not after 3 and 6 months [9]. Only one clinical study suggested a protective role of metformin on thyroid nodules growth in patients without insulin-resistance [10].

Taken together, these findings suggest that the long-term administration of metformin is able to lower serum TSH levels, selectively in patients with T2DM and hypothyroidism and in those with euthyroidism and TSH in the upper-normal quartile. Accordingly, these effects have been observed in another clinical model: patients with polycystic ovarian syndrome (PCOS) with hypothyroidism but not in euthyroid PCOS women [11, 12].

Several molecular mechanisms have been called into play to explain the TSH lowering effects of metformin. Some authors have suggested the increase of the central dopaminergic tone, the change of the affinity or the expression of thyroid hormone receptor or an effect on TSH regulation as possible explanatory mechanisms [13, 14].

TSH receptor (TSHR) is a protein made of two subunits, the *α* and the *β*, whose activation leads to the increase of intracellular adenylate cyclase levels. Follicle-stimulating hormone receptor (FSHR) and luteinizing-hormone receptor (LHR) share the same α subunit, and differ for the *β* subunit, which is receptor-specific. Molecular signaling of these three receptors is similar, since they belong to the G protein-coupled receptor family [15]. Interestingly, the existence of a cross-talk between the tyrosine kinase receptors [e.g., insulin receptor (IR), insulin-like growth one receptor (IGF1R)] and the G protein-coupled receptors has been demonstrated [16]. Specifically, an in-vitro study on mice granulosa cells showed that insulin receptor substrate 1 (IRS1) as the hub linking between the FSHR and the IGF1R-mediated activation of phosphatidylinositol 3-kinase (PI-3 K) [17]. More in detail, the incubation with FSH and the consequent FSHR-dependent increase of protein kinase A (PKA) activates the protein phosphatase 1*β* (PP1β), a ubiquitous eukaryotic Ser/Thr phosphatase, and the change in phosphorylation of specific domains of IRS1, thus leading to the IGF1R auto-phosphorylation [18] (Fig. 1, panel A). This pathway has also been confirmed in an experimental model of prepubertal porcine Sertoli cells [19]. Fascinatingly, patients with insulin-resistance show abnormally phosphorylated IRS1, which hinders the IR-dependent signaling cascade. The abnormal IRS1 phosphorylation may hypothetically interfere also with the signaling pathway of the G protein-coupled receptors, namely FSHR, LHR and TSHR. Accordingly, patients with insulin-resistance show a poorer response to FSH administration compared to patients with insulin-resistance but concomitantly treated with metformin [20]. We hypothesize that the abnormal phosphorylation of IRS1 occurring in patients with T2DM and/or insulin-resistance may somehow interfere with the TSHR signaling pathway (Fig. 1, panel B), thus inducing an increase of serum TSH levels. Treatment with insulin, by changing the IRS1 phosphorylation pattern, may sensitize the TSHR to TSH and this may explain the TSH lowering effects of metformin (Fig. 1). Encouragingly, the existence of a cross-talk also between the TSHR and the IGF1R has been recently shown [21] confirming that IRS1 is involved in the TSHR signaling. However, this needs to be validated by focused in-vitro studies.

**Fig. 1:**
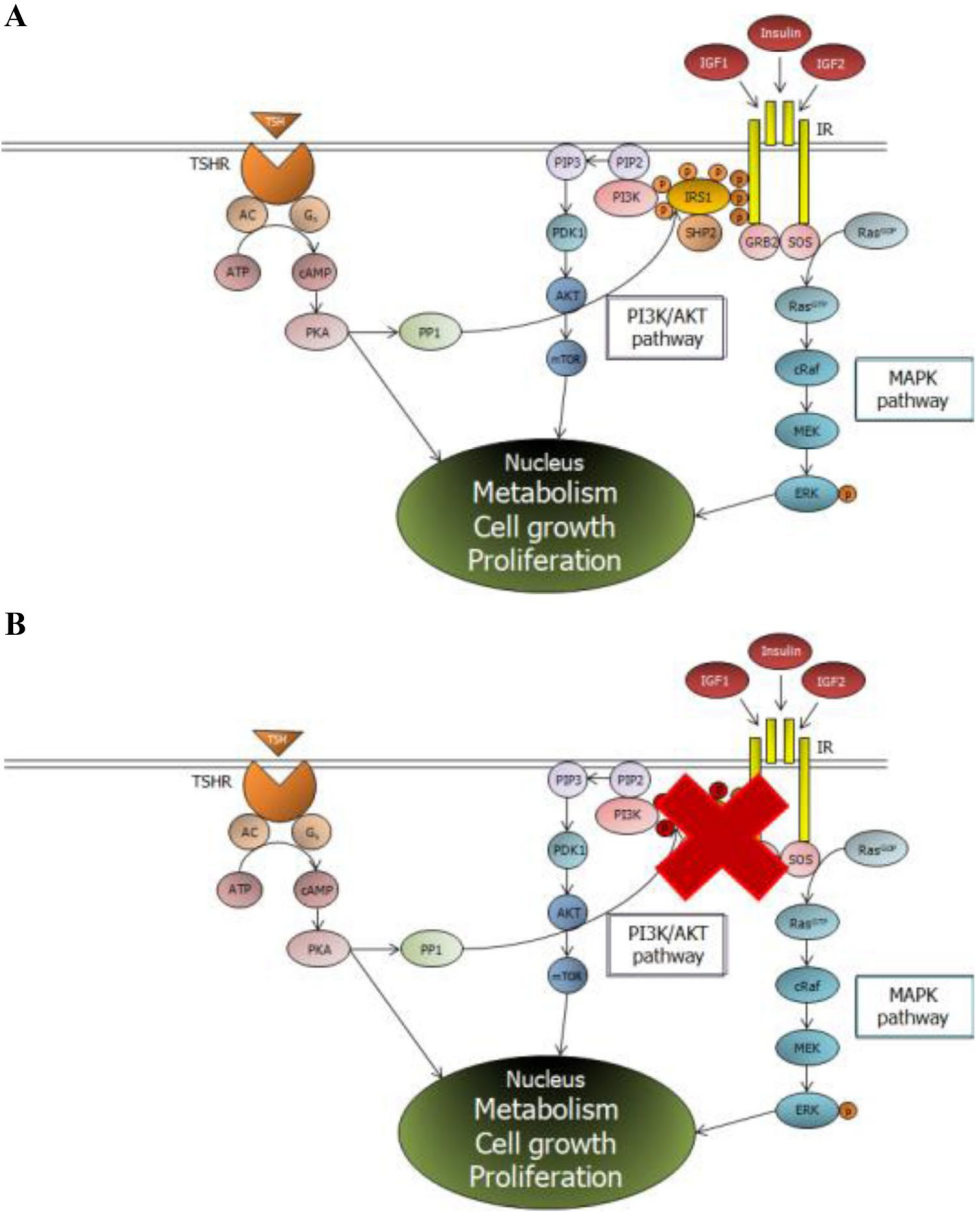
Proposed signaling pathway linking the Insulin receptor substrate 1 to thyroid stimulating hormone. **Panel A**. Thyroid stimulating hormone (TSH) by triggering its receptor (TSHR) increases intracellular cyclic adenosine monophosphate (cAMP) levels that, in turn, activates protein kinase A (PKA). Activated PKA phosphorylates the protein phosphatase 1β (PP1β), which modify the phosphorylation of specific domains of insulin receptor substrate 1 (IRS1), thus activating the phosphatidylinositol-3 kinase (PI3K)/protein kinase B (AKT) pathway. **Panel B** In patients with insulin-resistance, the abnormal pattern of IRS1 phosphorylation hinders the signaling pathway, partially interfering with the activation of the PI3K/AKT cascade

In conclusion, preliminary clinical evidence suggests that metformin has TSH lowering effects in patients with T2DM and hypothyroidism or in those with TSH serum levels in the upper normal value. Also, metformin may exert a protective role against thyroid nodules growth in patients without insulin-resistance. The cross-talk between tyrosine kinase receptors and the G protein-coupled receptors (which the TSHR belons to) has been already shown and IRS1 may represent the hub link between TSHR and IR pathways. By influencing IRS1 phosphorylation pattern, metformin may sensitize TSHR to TSH, thus explaining the findings of clinical studies. However, the existence of this molecular pathway must be confirmed through proper studies and further prospective randomized placebo-controlled studies are needed to confirm this hypothesis. Finally, due the impact of the tyrosine kinase receptor pathway (e.g., IGF1R) on thyroid nodules and cancer, the possible impact of metformin on thyroid suspicious nodules and cancer should be investigated, especially in the light of a recent in-vitro evidence showing a down-regulation of oncogenic genes in human anaplastic thyroid cancer cells after incubation with metformin and pioglitazone [22].
